# Preferred Communication Channels Among Older Adults During COVID-19

**DOI:** 10.1093/geroni/igab046.842

**Published:** 2021-12-17

**Authors:** Cheryl Der Ananian, Brad Doebbeling, G Mauricio Mejía, Hallie Wine, Michelle Houchins, Frank Infurna, Claire Pishko

**Affiliations:** 1 Arizona State University, Phoenix, Arizona, United States; 2 College of Health Solutions, Arizona State University, Phoenix, Arizona, United States; 3 Arizona State University, Tempe, Arizona, United States

## Abstract

Social distancing measures put in place during the COVID-19 pandemic limited in-person interactions and may have increased the risk for social isolation and loneliness in older adults. Purpose: The purpose of this study was to understand the communication channels used by older adults (age 50+) during the COVID-19 pandemic to mitigate social isolation and loneliness. Methods: Older adults (n=22) who were selected from a longitudinal study, ‘Aging in the time of COVID,’ and who had self-reported they successfully avoided loneliness, participated in a semi-structured online interview. Participants were asked to describe the communication techniques and efforts they used to stay connected to family and friends during the pandemic. All interviews were recorded and transcribed verbatim. A thematic analysis approach was used to identify common approaches. Results: Participants were primarily female and white (100%) with a mean age of 64.7 years. Preliminary findings (n=5) suggest older adults relied heavily on technology to facilitate communication with family and friends including texting, phone calls, email, video calls or conferences (e.g., Zoom), WhatsApp and social media, primarily Facebook. In-person communication strategies, including one-on-one and small group gatherings following social distancing guidelines, were preferred, but used less often than technology-based approaches. Living close to friends and family, and previous experience with technology were facilitators. Competing work and family demands, distance, and technology challenges limited communication. Conclusions: While older adults may adopt technology at a lower pace, they relied on digital communication technology to maintain social connections during COVID.

